# Digital clocks: simple Boolean models can quantitatively describe circadian systems

**DOI:** 10.1098/rsif.2012.0080

**Published:** 2012-04-12

**Authors:** Ozgur E. Akman, Steven Watterson, Andrew Parton, Nigel Binns, Andrew J. Millar, Peter Ghazal

**Affiliations:** 1 Centre for Systems, Dynamics and Control, College of Engineering, Computing and Mathematics, University of Exeter, Harrison Building, North Park Road, Exeter EX4 4QF, UK; 2 Division of Pathway Medicine, University of Edinburgh Medical School, Chancellor's Building, 49 Little France Crescent, Edinburgh EH16 4SB, UK; 3 SynthSys Edinburgh, University of Edinburgh, CH Waddington Building, King's Buildings, Mayfield Road, Edinburgh EH9 3JD, UK; 4 Department of Mathematics, University of Edinburgh, James Clerk Maxwell Building, King's Buildings, Mayfield Road, Edinburgh EH9 3JZ, UK

**Keywords:** systems biology, circadian gene networks, Boolean logic, photoperiodism, *Arabidopsis thaliana*

## Abstract

The gene networks that comprise the circadian clock modulate biological function across a range of scales, from gene expression to performance and adaptive behaviour. The clock functions by generating endogenous rhythms that can be entrained to the external 24-h day–night cycle, enabling organisms to optimally time biochemical processes relative to dawn and dusk. In recent years, computational models based on differential equations have become useful tools for dissecting and quantifying the complex regulatory relationships underlying the clock's oscillatory dynamics. However, optimizing the large parameter sets characteristic of these models places intense demands on both computational and experimental resources, limiting the scope of *in silico* studies. Here, we develop an approach based on Boolean logic that dramatically reduces the parametrization, making the state and parameter spaces finite and tractable. We introduce efficient methods for fitting Boolean models to molecular data, successfully demonstrating their application to synthetic time courses generated by a number of established clock models, as well as experimental expression levels measured using luciferase imaging. Our results indicate that despite their relative simplicity, logic models can (i) simulate circadian oscillations with the correct, experimentally observed phase relationships among genes and (ii) flexibly entrain to light stimuli, reproducing the complex responses to variations in daylength generated by more detailed differential equation formulations. Our work also demonstrates that logic models have sufficient predictive power to identify optimal regulatory structures from experimental data. By presenting the first Boolean models of circadian circuits together with general techniques for their optimization, we hope to establish a new framework for the systematic modelling of more complex clocks, as well as other circuits with different qualitative dynamics. In particular, we anticipate that the ability of logic models to provide a computationally efficient representation of system behaviour could greatly facilitate the reverse-engineering of large-scale biochemical networks.

## 1. Introduction

Circadian rhythms are the fundamental daily oscillations in metabolism, physiology and behaviour that occur in almost all organisms, ranging from cyanobacteria to humans [1]. The gene regulatory networks (GRNs), or *clocks*, that generate these rhythms regulate the expression of associated genes in roughly 24-h cycles. Circadian networks have been studied in a variety of experimentally tractable model systems, revealing that different organisms share structurally similar circuits based around interlocking sets of positive and negative gene–protein feedback loops [2–4]. In mammals, circadian rhythms are being increasingly recognized as important to healthy phenotypes, playing a role in ageing [5], cancer [6], vascular disease [7] and psychiatric disorders [8], as well as modulating innate immunity [9–11].

Clocks synchronize to their environment by using light and temperature to regulate the levels of one or more components of the feedback loops. This ensures that key biological processes are optimized relative to dawn and dusk, benefiting growth and survival [12,13]. For the clock to provide such an adaptive advantage, the phase must change appropriately when the clock is subject to regular perturbations—particularly seasonal changes in daylength (the *photoperiod*). However, as well as exhibiting flexible responses to variations in the input light signal, the clock must also exhibit robustness to irregular perturbations, such as genetic mutations and the intrinsically stochastic environment of the cell.

Temperature also plays a critical role as an environmental time cue. Across different species, the clock is relatively insensitive to temperature in that the period of free-running oscillations typically has a *Q*_10_ value close to 1 [14–16]. This latter phenomenon, known as temperature compensation, is generally considered to be one of the defining properties of the circadian clock and has been suggested to be a key requirement for stability of the clock's phase relationship under seasonal temperature variations [17,18].

The ability of ordinary and delay differential equations (DEs) to reproduce the underlying continuous dynamics of biochemical networks, and to parametrize individual reactions has led to the construction of DE models in a number of circadian organisms. These include the fungus *Neurospora crassa* [19–25], the fly *Drosophila melanogaster* [26–29], the mammal *Mus musculus* [30–33] and the higher plant *Arabidopsis thaliana* [34–38]. Such models have proved useful in uncovering the general design principles of circadian oscillators, as well as providing a quantitative framework within which to interpret experimental results [4,38]. In particular, novel insights have been gained into the mechanisms promoting robustness with respect to photoperiod changes [25], temperature fluctuations [18,23] and molecular noise [39–41]. The DE models have also yielded experimentally testable predictions that have led to the discovery of novel circadian regulators [36].

However, a significant drawback of the DE approach is that the values of the kinetic parameters controlling each individual reaction have to be specified, and for clocks these are typically unknown. When constructing a DE model, it is therefore necessary to calculate the particular combination of parameter values giving an optimal fit to experimental data [18,25,34–37]. For realistic systems involving large numbers of reactions, this optimization procedure is computationally very expensive making exhaustive parameter searches intractable. With increasing parameter numbers also comes a need for data with which to constrain the optimization, placing a greater demand on experiment in terms of finance, time and ethics. These concerns mean that there is a pressing need for modelling approaches that minimize the number of parameters required, while adequately capturing the essential dynamical behaviour of the system of interest.

Here, we develop just such an approach, based on Boolean logic. In Boolean models, the activity of each gene is described with a two-state variable taking the value ON (1) or OFF (0), meaning that its products are present or absent, respectively. Biochemical interactions are represented by simple, binary functions that calculate the state of a gene from the activation state of its upstream components [42–50]. This approximation dramatically reduces the state–space of the system, mapping the infinite number of different continuous system states in a DE model to a finite number of discrete states in the Boolean equivalent.

An additional important advantage of using a logic approach is that the total number of parameters is substantially reduced. For a given gene, the full set of reactions determining its state through a particular interaction is parametrized by a single *signalling delay*, representing the net time taken for these reactions to cause a change in state. Fitting to experimental data introduces an associated *discretization threshold*, with expression levels above the threshold taken to correspond to the ON state of the gene and levels below it to the OFF state [46–48,50]. In fitting a particular experimental dataset, each delay becomes a multiple of the sampling interval, while only a bounded subset of thresholds will yield distinct Boolean expression patterns. This means that the total number of parameter combinations is finite and can be enumerated. Thus, by building a logic version of a DE model, an infinite model can be converted into a finite one with fewer parameters to be optimized. This extends the scale and complexity of GRNs that can be studied by Boolean models far beyond the practical scope of DEs.

In this work, we introduce the first Boolean models of circadian networks. By constructing logic analogues of a number of established DE clock models, we demonstrate that in each case the Boolean models are capable of accurately reproducing the higher-order properties—particularly photoperiod responses—of their DE counterparts. This suggests that the complex, biological signal transduction simulated by the DE models can be captured in Boolean equivalents possessing significantly smaller parameter sets. We introduce a general method for optimizing Boolean models that avoids the qualitative and often subjective terms characteristic of the cost functions used to fit the parameters of large DE clock models. Furthermore, we show that our fitting algorithm is capable of determining the optimal Boolean model configuration associated with a given circuit topology and experimental dataset. In particular, our algorithm successfully predicts the recently discovered repressive action of the circadian gene TOC1 on LHY in the central feedback loop of the *Arabidopsis* clock [51].

Taken together, our results show that Boolean models can quantitatively distinguish between a range of putative regulatory structures on the basis of the system dynamics. This identifies Boolean logic as a viable technique for reverse-engineering circadian networks, complementing approaches based on DEs. Moreover, our work also suggests novel hybrid modelling approaches based on employing Boolean models as a first step towards the construction of more detailed DE formulations. More generally, we propose that our methodology provides an efficient way of systematically modelling complex signalling pathways, including other oscillatory circuits and systems characterized by steady-state dynamics.

## 2. Results

### 2.1. Logic models employ significantly fewer parameters

**Figure 1.**
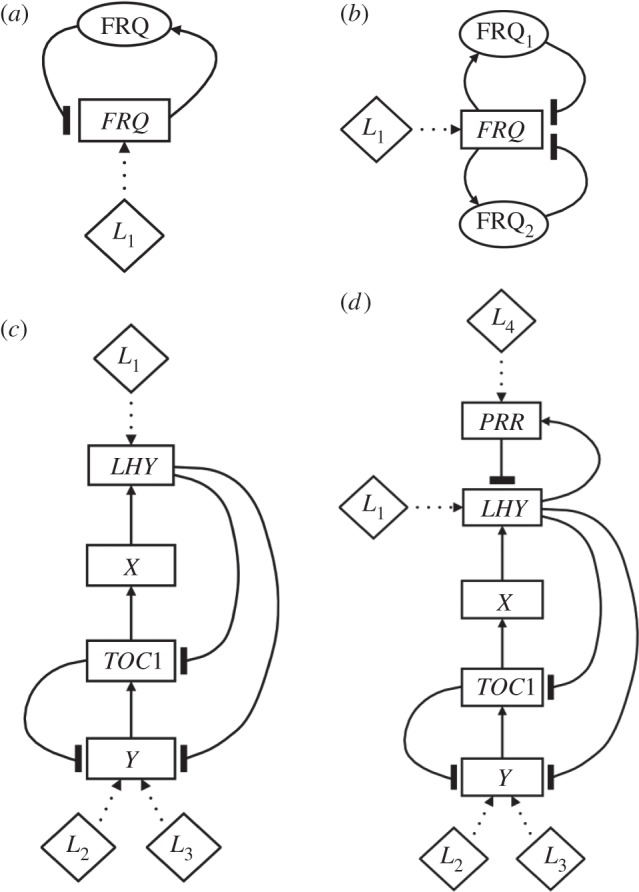
Circuit diagrams for the clock models. Genes are boxed and arrows denote regulatory interactions. Diamonds represent light inputs. (*a*) The single-loop *Neurospora* model [19]. FRQ protein represses production of *frq* transcript. Light acts on the network by upregulating *frq* transcription. (*b*) The two-loop *Neurospora* model [18]. Two isoforms of FRQ are produced which both repress *frq* transcription. Light upregulates *frq* as in diagram (*a*). (*c*) The two-loop *Arabidopsis* model [35]. TOC1 activates its repressor LHY (combining LHY and CCA1) indirectly through a hypothetical gene X, forming the central negative feedback loop of the circuit. LHY is directly upregulated by light while light indirectly activates TOC1 via a second hypothetical gene Y, posited to have two distinct light inputs. Y activates TOC1 transcription and TOC1 represses Y, forming a second, interlocked feedback loop. (*d*) The three-loop Arabidopsis model [36]. The additional PRR gene (combining PRR7 and PRR9) is light-activated and represses LHY transcription. LHY upregulates PRR, creating a third feedback loop.

We selected four recent circadian oscillator models of increasing complexity with which to assess the suitability of a Boolean formulation. The simplest of these was a *Neurospora* model based on a single negative feedback loop with a single light input [19] (figure 1*a*). This is represented by three DEs parametrized by 13 kinetic constants. The second model was a modified version of the 1-loop *Neurospora* circuit in which there is a pair of negative feedback loops associated with different isoforms of the active protein [18] (figure 1*b*). The extra feedback loop results in five DEs parametrized by 18 kinetic constants. The third model was an *Arabidopsis* circuit based on a pair of interlocking feedback loops with three light inputs [35] (figure 1*c*). It is described by 13 DEs together with 64 kinetic constants. The final model considered was a 3-loop *Arabidopsis* circuit obtained by adding an extra feedback loop and light input to the 2-loop system [36] (figure 1*d*). This yields 16 DEs parametrized by 80 kinetic constants.

In a Boolean model, an interaction, *j*, between two components *X*_*i*_ and *X*_*k*_ is quantified by the corresponding signalling delay *τ*_*j*_. This is the time taken for the biochemical processes represented by *j* to convert a change in the state of *X*_*i*_ into a change in the state of *X*_*k*_ (electronic supplementary material, figure S1). The signalling delays are thus parameters that determine the dynamics of the model, with different combinations of delays yielding different attractor states (e.g. steady-states or limit cycles) [43,44,52,53]. In order to relate the discrete dynamics to the continuous variations in expression level observed experimentally, it is necessary to introduce a discretization process. A hypothetical time course is shown in the electronic supplementary material, figure S2*a*; and the result of applying a particular discretization threshold is shown in the electronic supplementary material, figure S2*b*. In the general case, the choice of threshold is dependent on the effective range of activity and expression that is critical for signal propagation. As a result, each threshold also becomes a parameter of the logic model [46,47,50].

**Figure 2.**
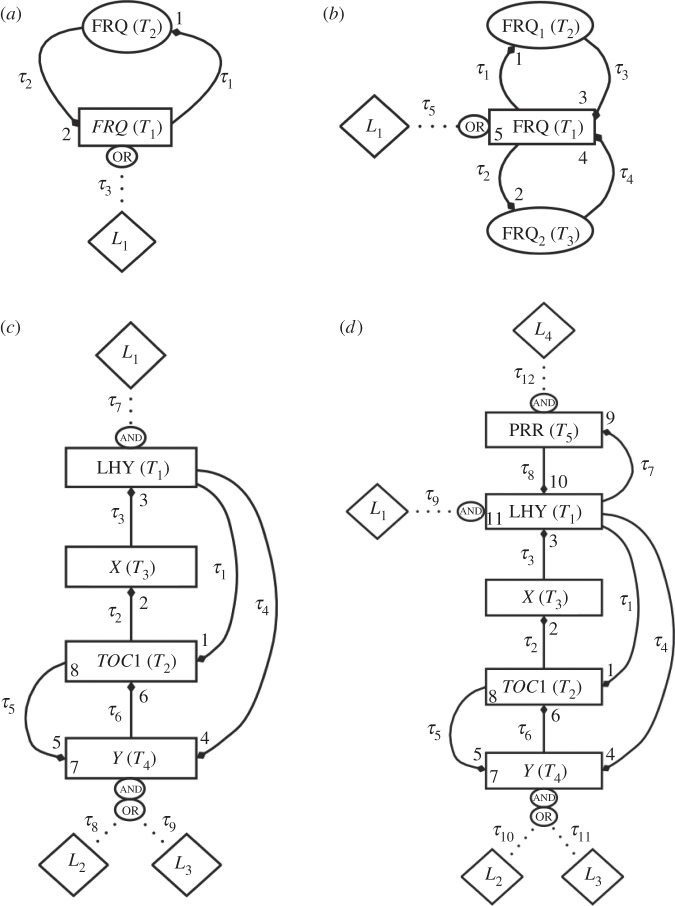
The abstract topologies for the logic representations of the clock circuits shown in figure 1. Genes are boxed and arrows denote regulatory interactions. (*a*) The single-loop *Neurospora* model. (*b*) The 2-loop *Neurospora* model. (*c*) The 2-loop *Arabidopsis* model. (*d*) The 3-loop *Arabidopsis* model. Numerals *l* index logic gates *g*_*l*_ ∈ {0,1} that can be varied to generate different regulatory structures. Numerals at the end of an arrow index the single-input gate defining that interaction. Numerals within boxes index logic gates governing double-input interactions. Diamonds represent light inputs, with the corresponding fixed gates in ovals indicating how these affect the target species (e.g. in (*c*), *L*_2_ and *L*_3_ are combined with an OR gate after which the resulting bitstring is combined with the output of Y through an AND gate; see §4 for full details). *τ*_*j*_s represent the circuit delays and *T*_*i*_s the discretization thresholds used to fit continuous data.

**Figure 3.**
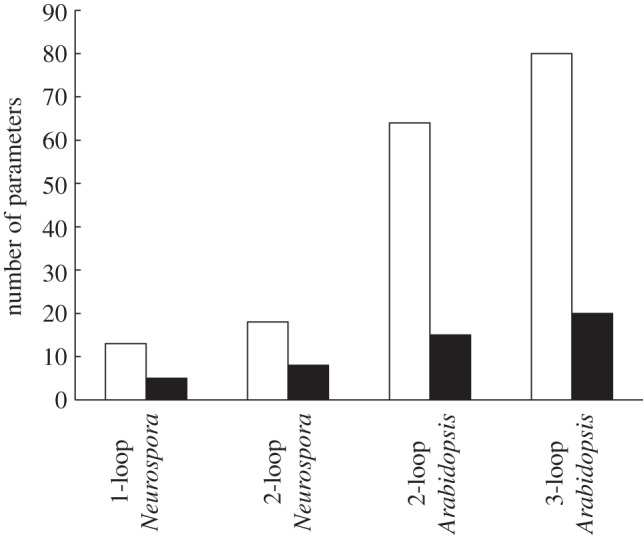
The number of parameters required for each clock configuration as DE models (white bars) and logic models (black bars).

For logic descriptions of the circadian networks shown in figure 2, each edge is parametrized by a signalling delay *τ*_*j*_, and each vertex by a threshold *T*_*i*_. Thus, a network is characterized by the vectors *τ* = (*τ*_*j*_) and **T** = (*T*_*i*_). In figure 3, we compare the total number of parameters in the logic and DE versions of each network (see also the electronic supplementary material, table S1). We can see that dramatically fewer parameters are required in a logic description compared with the corresponding DE formulation. Indeed for the largest network considered, 3-loop *Arabidopsis*, the Boolean description reduces the number of parameters by a factor of 4.

### 2.2. Logic model configurations consistent with differential equation models are optimal

In identifying the logic models that best reproduce the corresponding DE dynamics, we considered variations to the structures of the networks shown in figure 1. Starting from the abstract topologies of figure 2, each edge is activating or inhibiting, and where two or more edges lead into a vertex, the corresponding inhibition/activation signals are combined to determine the state of the gene.

In the Boolean formalism, the manner in which regulatory signals affect a gene's expression level is represented by the corresponding *logic gate*. This is a binary function that specifies the current state of the gene, ON (1) or OFF (0), for each possible combination of input states [46,54]. For genes with a single input, there are two possible functions for which the output varies with the input: the identity gate, in which the output follows the input (0 → 0 and 1 → 1); and the NOT gate, in which the input is inverted (0 → 1 and 1 → 0). These represent activation and repression of the gene by its regulator, respectively. Genes with two inputs are commonly modelled using either an AND or an OR gate [46,49,54]. For simplicity, we considered each multi-input gate to be a composition of ANDs and ORs (see §4 for further details). The particular combination of logic gates used to model a network is referred to here as its *logic configuration* (*LC*): this encodes the regulatory structure of the network in a compact fashion.

It follows that each abstract topology of figure 2 gives rise to 2^*E+M*^ possible LCs, where *E* is the number of edges, and *M* is the number of vertices with more than one input. The total number of possible LCs is 4 for 1-loop *Neurospora*, 32 for 2-loop *Neurospora*, 256 for 2-loop *Arabidopsis* and 2048 for 3-loop *Arabidopsis*. In each case, a subset of these LCs is consistent with the pattern of activation and inhibition in the corresponding DE model. That there can be more than one such LC in each case is due to the choice of AND or OR where a vertex has multiple edges leading into it. For *Neurospora*, the 1-loop circuit has a unique LC consistent with the DE model, whereas for the 2-loop circuit, there are two DE LCs. The 2- and 3-loop *Arabidopsis* networks yield four and eight DE LCs, respectively.

For a given logic model, we would expect the DE LCs to most closely reproduce the DE dynamics. To test this hypothesis, we optimized LCs to synthetic experimental data obtained from the DE system, and then compared their predictive performance. For each LC, the match to the continuous dynamics was quantified by finding the combinations of parameters (signalling delays and discretization thresholds) that minimized a quantitative cost function. In order to be able to objectively compare the ability of the Boolean models to reproduce experimental time courses against that of their DE counterparts, we employed a cost function that closely mirrored those commonly used to optimize continuous models [18,25,34–36,38]. The cost function we used measured the goodness-of-fit of each logic model to synthetic data generated in both 24 h light–dark (LD) cycles and the appropriate free-running light regime (continuous dark, DD, for *Neurospora*; continuous light, LL, for *Arabidopsis*). At each vertex, the cost score was calculated as the correlation between the discretized time series for the downstream species and the time-delayed predicted output calculated from the discretized data for the upstream species. Scores were summed across all vertices and light regimes to give the final cost value (see §4 for details).

After ranking the optimized LCs by score, we then assessed whether the top-ranking LCs comprised viable clock circuits by checking that they were capable of (i) generating self-sustained oscillations with a circadian period in constant conditions and (ii) entraining to LD cycles over a realistic range of photoperiods [55].

**Figure 4.**
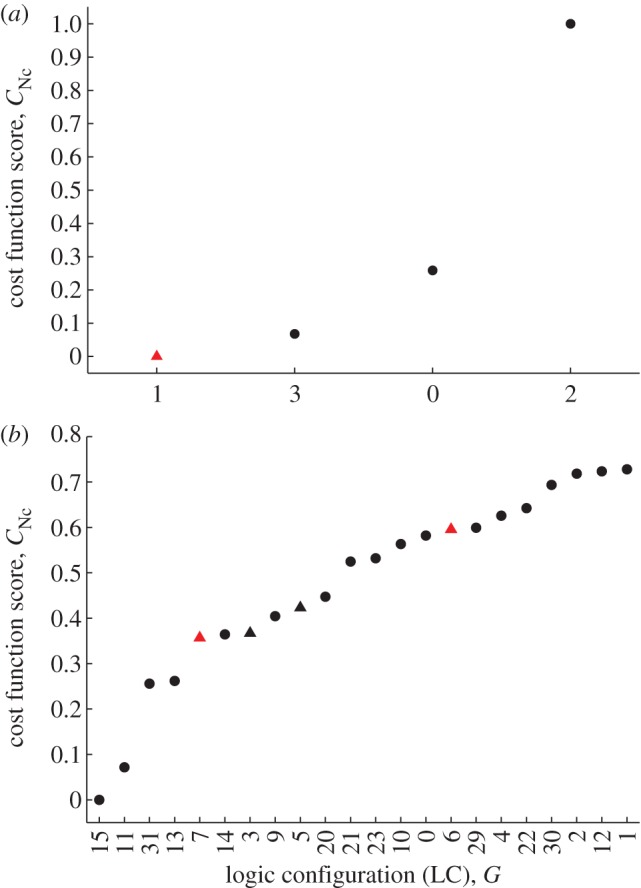
The results of exploring the logic configurations (LCs) belonging to the abstract topologies of the (*a*) 1-loop and (*b*) 2-loop *Neurospora* models. Cost scores are shown for the optimal fit of each LC to synthetic data. The LCs are indexed by their decimal representations for brevity (see §4 for details). Here, a score of 0 indicates the best fit and a score of 1 the worst fit. Triangles indicate LCs for which the Boolean model yields a viable clock. LCs mirroring the activation and inhibition pattern of the corresponding DE models in figure 1*a*,*b* are plotted in red. In (*a*), one such LC mirrors the corresponding DE model, *G* = (01), and this emerges as the optimal configuration yielding a viable clock. In (*b*), only LCs yielding scores less than 0.75 are shown. There are two that mirror the equivalent DE model. One of these, *G* = (00111), is identified as the optimal configuration giving a viable clock (leftmost red triangle). In this LC, either of the FRQ isoforms can independently inhibit transcription (the corresponding two-input gate is of the AND type).

**Figure 5.**
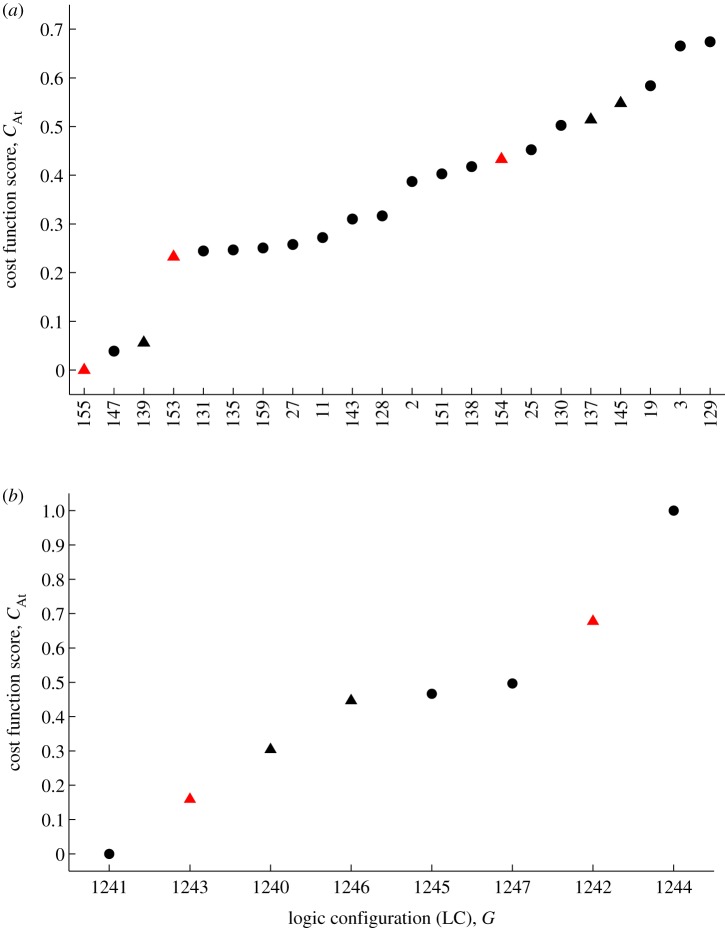
The results of exploring the logic configurations (LCs) belonging to the abstract topologies of the (*a*) 2-loop and (*b*) 3-loop *Arabidopsis* models. Cost scores are shown for the optimal fit of each LC to synthetic data. As in figure 4, each LC is indexed by its decimal expansion. Scores of 0 and 1 indicate the best and worst fits, respectively. Triangles denote LCs for which the Boolean model yields a viable clock. LCs mirroring the activation and inhibition pattern of the corresponding DE models in figure 1*c*,*d* are plotted in red. In (*a*), only LCs yielding scores less than 0.75 are shown. Of these, there are three LCs consistent with the activation and inhibition pattern of the equivalent DE model from a possible total of four. From this subset, *G* = (10011011) emerges as the optimal configuration yielding a viable clock. For this circuit, both LHY and TOC1 can independently inhibit Y, while TOC1 is repressed unless LHY is repressed while Y is activated (the corresponding gates are both of the AND type). In (*b*), only the eight LCs obtained by varying the gates of the PRR–LHY loop were considered: all other gates were fixed to those of the optimal 2-loop configuration. Of these eight possible circuits, two LCs were consistent with the equivalent DE model, from which *G* = (10011011011) is identified as the optimal configuration giving a viable clock. This LC corresponds to a clock network in which LHY is repressed unless PRR is inactive and X is active (the corresponding gate is of the AND type).

For the *Neurospora* and 2-loop *Arabidopsis* logic models, the full set of LCs was fitted to synthetic data. The best-performing LCs for these networks are shown in figures 4 and 5*a*. It can be seen that the optimization method produces a clear separation of the LCs by score. Moreover, for each network, one of the DE LCs is uniquely identified as the optimal circuit yielding a viable clock. In the case of 3-loop *Arabidopsis*, we considered the subset of configurations obtained by setting the gates common with the 2-loop *Arabidopsis* circuit to their optimized values. This mirrored the construction of the 3-loop DE model which was derived from the 2-loop system by adding an additional feedback loop while fixing all other interactions, and then optimizing the parameters of the new loop [36]. Constraining the structure of the circuit in this fashion yielded eight possible LCs (the two edges in the LHY–PRR loop described as activation or inhibition and the AND/OR interaction at LHY; figure 2*d*). Figure 5*b* shows that in this system, as for the other models, a DE LC emerges as the optimal circuit.

### 2.3. Optimal Boolean models have biological time-series characteristics

**Figure 6.**
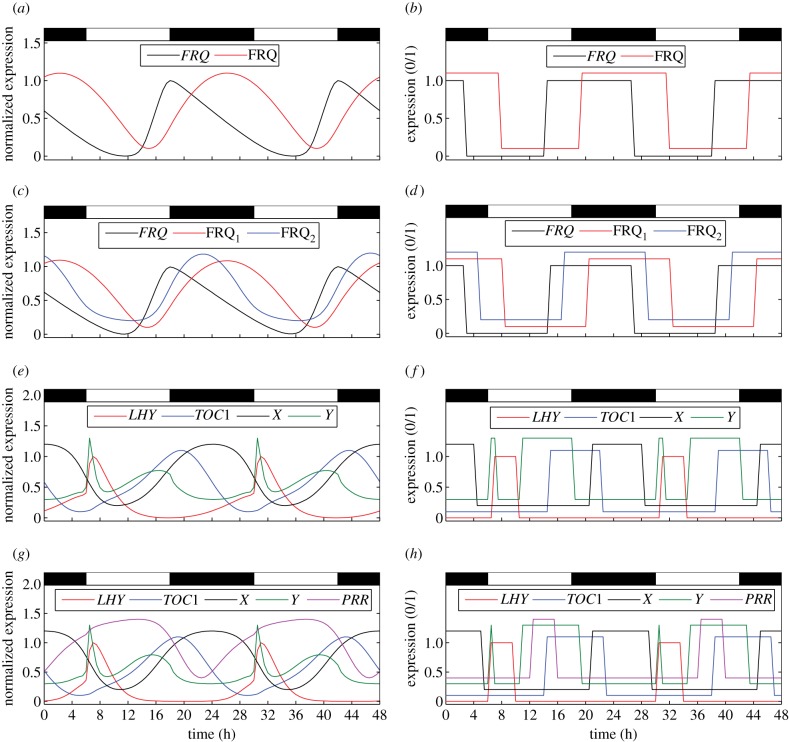
Time series for the differential equation and Boolean versions of the clock models in 12:12 LD cycles. Two 24-h cycles are plotted for each model. (*a*,*b*) 1-loop *Neurospora*; (*c*,*d*) 2-loop *Neurospora*; (*e*,*f*) 2-loop *Arabidopsis*; (*g*,*h*) 3-loop *Arabidopsis*. Differential equation time series (left panels) have been normalized to lie between 0 and 1 in order to facilitate comparison with the Boolean simulations (right panels). Different components within a model are slightly offset from one another so they can be distinguished more easily. The time step used for solving the Boolean models was 0.5 h, equal to the data sampling interval.

The time series generated by the optimal configurations in LD cycles are shown in figure 6. The corresponding DE simulations are also plotted for comparison.

In each case, it is clear that the Boolean models capture the same qualitative dynamics as their DE counterparts. Different species are switched on and off relative to one another with phases that match the patterns of rising and falling expression in the corresponding continuous time series. Moreover, the delays between the switching times are similar to the phase differences between the peaks and troughs of the DE solutions.

It should be noted that both Boolean *Arabidopsis* models reproduce the acute light response in the Y gene, as well as the circadian response in Y around dusk (cf. figure 6*e*–*h*). This demonstrates the ability of the Boolean circuits to simulate biochemical processes that occur on different time scales within the same system.

The optimal LCs give equally good matches to the DE dynamics in simulated free-running conditions, as can be seen in the electronic supplementary material, figure S3.

### 2.4. Optimal Boolean models have biological photoperiodic behaviour

In order to assess the extent to which the Boolean models reproduce the DE dynamics in a more global and quantitative fashion, we compared phase changes over a range of biologically realistic photoperiods. In the Boolean framework, the times at which solutions switch between 0 and 1 emerge as natural phase measures. For continuous time courses—such as those generated by DE models—the point in the circadian cycle at which the expression level of a molecular species decreases below a set threshold can be employed as a phase marker [25,56,57]. This suggested using the time at which each species decreases below its discretization threshold as a phase measure for the DE simulations, and the time of the 1 → 0 transition as the equivalent marker in the Boolean models.

**Figure 7.**
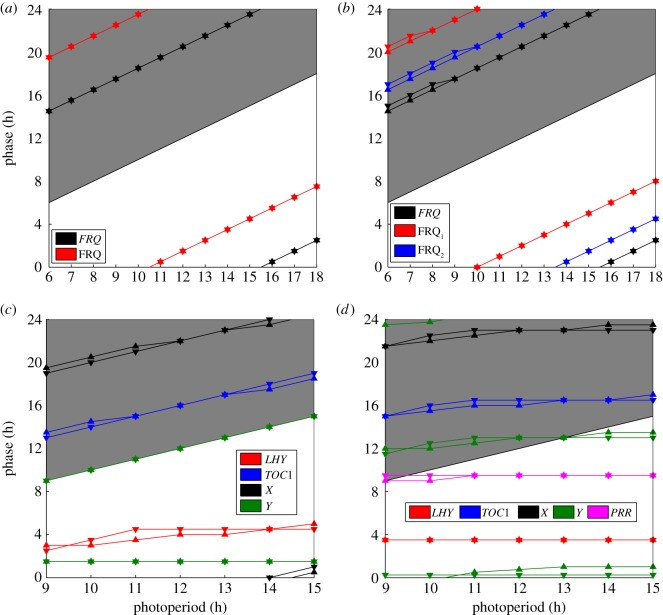
Comparing the photoperiodic behaviour of the Boolean and differential equation (DE) versions of each model. For Boolean models, the phase of each species is taken as the time within the LD cycle of the ON to OFF transition (downward triangles). Analogously, the DE model phases are defined as the times at which species decrease below the thresholds yielding the optimal fit of the corresponding Boolean circuit to data (upward triangles). Shaded areas of plots, darkness; open areas, light. (*a*) 1-loop *Neurospora*. The phase–photoperiod profiles are coincident, indicating that the Boolean model exactly reproduces the photoperiodic behaviour of its DE counterpart: FRQ transcript and protein are both locked to dusk across the photoperiod range. (*b*) 2-loop *Neurospora*. The phase plots are almost exactly equal, except for shorter photoperiods where they differ by the data sampling interval. As for (*a*), all components are locked to dusk. (*c*) 2-loop *Arabidopsis*. The Boolean and DE models exhibit very similar patterns of dawn- and dusk-locking across genes. The two Y phase–photoperiod profiles reflect the double peak observed in this component (figure 6*e*) which gives rise to a (dawn-locked) light-induced peak and a (dusk-locked) circadian peak [57]. (*d*) 3-loop *Arabidopsis*. The phase plots are similar, with all components predominately dawn-locked. As for 2-loop *Arabidopsis*, the two Y profiles reflect the double peak observed for this gene (figure 6*g*).

The phase–photoperiod relationships computed in this fashion are shown for the *Neurospora* models in figure 7*a*,*b*. For both networks, the photoperiodic behaviour of the Boolean and DE models is very close: indeed for the 1-loop network, they are exactly equivalent. Figure 7*c*,*d* plots the photoperiod simulations obtained with the *Arabidopsis* circuits. Here too, the phase–photoperiod profiles are very similar, with the addition of the LHY–PRR loop to the 2-loop model causing a transition from a predominately dusk-locked system to a dawn-locked one [57]. In particular, the Boolean 2-loop *Arabidopsis* circuit exactly reproduces the dual light response in the Y gene, in which the acute peak tracks dawn, and the circadian peak tracks dusk. This suggests that the logic circuits possess sufficient dynamic flexibility to perform the complex integration of environmental signals that is a central property of circadian systems.

### 2.5. Boolean models can determine circadian network structure from experimental data

The success of the logic models in recovering the correct DE configurations from synthetic data suggested that for a fixed abstract topology, our optimization procedure has the capacity to determine the logic network most consistent with a given dataset. We tested this finding further by optimizing the 3-loop *Arabidopsis* logic circuit to highly sampled experimental time series recorded using luciferase (LUC) imaging in constant light from a wild-type strain [57]. All possible LCs were considered, corresponding to a network inference carried out assuming no prior biological knowledge. The cost function optimized was the same as that used for fitting to synthetic LL data. As previously, viable clock circuits were taken to be those yielding autonomous limit cycles with circadian periods.

**Figure 8.**
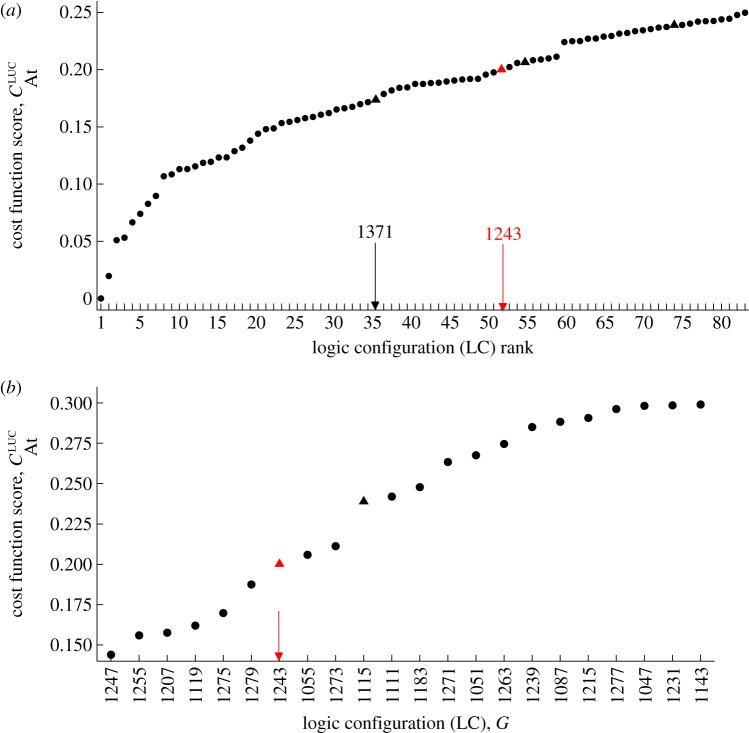
Identifying the logic configurations (LCs) of the 3-loop *Arabidopsis* model giving the best fits to experimental data. Plotting conventions are as described in figures 4 and 5. (*a*) Top-ranking LCs. The black arrow indicates the optimal configuration yielding a viable clock, *G*_OPT_ = (10101011011). For this circuit, LHY and TOC1 repress each other, in agreement with recent biochemical evidence [51]. The red arrow denotes the second highest-ranking viable LC, *G*_DE_ = (10011011011). This LC matches the regulatory structure of the DE model, and was previously identified as the configuration yielding the best fit to synthetic data (figure 5*b*). (*b*) Top-ranking LCs obtained under the constraint that LHY represses TOC1 and TOC1 activates LHY (i.e. *g*_1_ = 1 and *g*_2_ = *g*_3_ = 0; figure 2*d*). Under this assumption, *G*_DE_ emerges as the optimal clock circuit. The circuit diagrams for *G*_OPT_ and *G*_DE_ can be seen in the electronic supplementary material, figure S4.

The results of fitting to experimental data are presented in figure 8*a*. It can be seen that the second highest-ranking LC giving a viable circuit is a DE configuration. This configuration, *G*_DE_, is in fact the same as that previously determined to be optimal from the synthetic *Arabidopsis* datasets. Moreover, figure 8*b* shows that *G*_DE_ emerges as the top-ranking clock configuration if the regulatory structure is constrained to incorporate the central LHY–TOC1 negative feedback loop of the corresponding continuous model. Interestingly, the optimal configuration, *G*_OPT_, differs from *G*_DE_ in the sign of the TOC1–LHY interaction, with TOC1 repressing LHY in *G*_OPT_ as opposed to activating it (compare electronic supplementary material, figure S4*a*,*b*). This result is consistent with the recent experimental characterization of the LHY–TOC1 circuit as a double-negative feedback loop, rather than the single-negative loop assumed in the 2- and 3-loop DE models [51].

**Figure 9.**
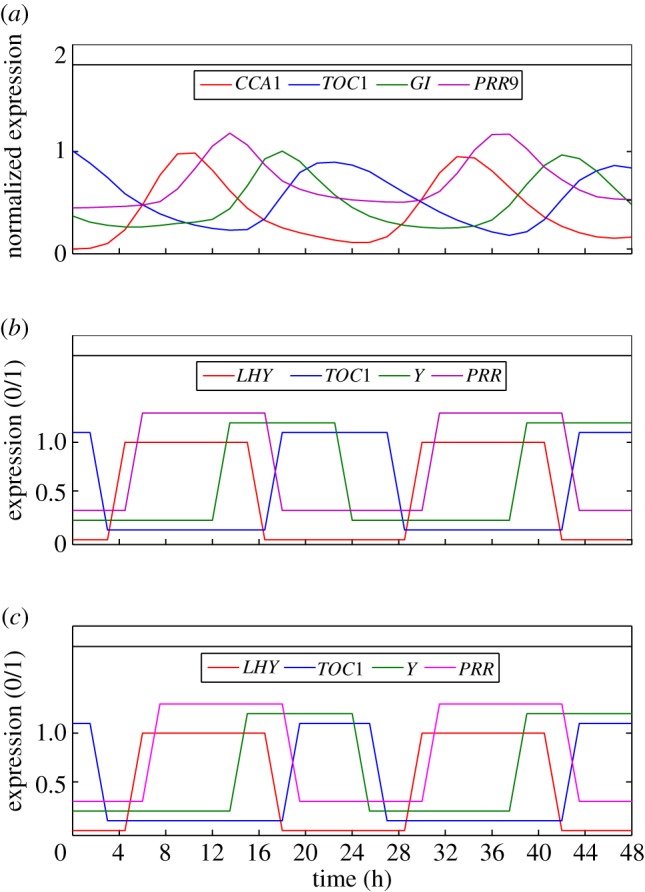
Simulations generated by the optimal fits of the 3-loop *Arabidopsis* model to experimental data. (*a*) Experimental expression profiles for the genes CCA1, TOC1, GI and PRR9 in free-running conditions (LL). Expression levels were determined using LUC reporter gene imaging constructs and have been normalized to lie between 0 and 1. (*b*) The equivalent Boolean time series generated by the logic configuration *G*_OPT_ yielding the best fit to data. (*c*) Boolean expression profiles for the highest-ranked configuration *G*_DE_ incorporating the central LHY–CCA1 negative feedback loop of the DE model. In all plots, different components are slightly offset from one another so they can be distinguished more easily. The time step used for solving the logic model was 1.5 h, equal to the data sampling interval.

Figure 9*b*,*c* shows the simulations of the free-running clock obtained from the optimal and DE configurations, respectively. For both LCs, the simulated oscillation period and timing of gene expression are close to that of the natural system (cf. figure 9*a*). This can be seen clearly by comparing the durations for which each gene is ON in the two Boolean models with the corresponding peaks in the continuous data.

## 3. Discussion

### 3.1. A new approach to quantitative circadian modelling based on Boolean logic

Circadian clocks have become popular systems for studying the relationship between gene–protein dynamics and phenotype. The molecular machinery underlying these networks has been relatively well characterized, and this has led to great interest in developing predictive computational models of the clock. Such models are usually formulated as sets of DEs. The high level of biochemical detail afforded by this approach has allowed DE models to successfully address a range of issues regarding the functional relationship between the architecture of the clock and circadian homeostasis. One homeostatic mechanism that has been comprehensively investigated from a theoretical perspective is temperature compensation. By assuming that certain subsets of a circuit's kinetic parameters are temperature-dependent, temperature has been incorporated into a number of circadian DE models, ranging from minimal circuits built on the Goodwin oscillator [15,23,58] to more detailed formulations that explicitly model the underlying biochemical reactions [18,24]. These models have provided new insights into the possible mechanisms that lead to circadian mutants with altered compensation properties, as well as suggesting generic motifs that may facilitate the tuning of the phase–temperature relationship.

However, clock models based on DEs are characterized by large numbers of kinetic parameters, the values of which are typically unknown. Optimizing these to experimental data is a major computational bottleneck, which imposes a hard bound on the maximum system size that can be studied. In addition, as the fitting problem is typically highly underdetermined and involves noisy, undersampled experimental data, robust optimization can require the construction of cost functions targeting specific qualitative features of the data, introducing a degree of arbitrariness to the fitting procedure [18,25,34–36].

The need for minimal clock parametrizations to address these issues has been recognized elsewhere in the literature. For example, recent *Neurospora* work has shown that it is possible to use a simple two-parameter function to represent all the intermediate processes between the expression of a clock gene and its action on a downstream target, while still maintaining sufficient flexibility to accurately simulate biological temperature and photoperiod responses [18,25].

An alternative technique for modelling GRNs is provided by Boolean logic. By assuming discrete expression levels, Boolean models provide an even greater reduction in complexity, albeit at the cost of reduced biochemical precision. Previous studies have exploited this reduction to study GRN state–space structures [48,59,60], and to introduce probabilistic models that parametrize statistical transitions between states [45,61]. The different limit cycles enumerable by a single Boolean GRN have been interpreted in some models as examples of cell differentiation [62,63], making limit cycle properties of special interest. Elsewhere, Boolean studies have focused on the regulatory logic underlying transcription [64–66], the immune response [44,53] and apoptosis [49].

Here, we have presented the first models of biological clocks based on the Boolean formalism. We constructed logic versions of four DE models simulating circadian rhythms in the organisms *N. crassa* and *A. thaliana*. To derive the best logic representations, both regulatory structure (LC) and parameters (signalling delays and discretization thresholds) were optimized to synthetic experimental data generated from the DEs. In each case, we found that the LC yielding the best fit to data was consistent with that of the corresponding DE system. This suggested that the Boolean models were capable of identifying the particular combination of activating and inhibiting elements best able to account for a set of experimental expression patterns (figures 4 and 5). We confirmed this hypothesis by successfully applying our optimization algorithm to real *Arabidopsis* LUC data (figure 8). These results demonstrate that the logic models possess sufficient predictive power to perform biological network inference, despite their relative simplicity. Moreover, the optimization to LUC data highlights the importance of network structure in determining dynamic behaviour [67,68]. This optimization gave rise to a pair of putative 3-loop network architectures with distinct parameter values that generated very similar expression time series (cf. figure 9*b*,*c* and electronic supplementary material, figure S4). One of these architectures matched the 3-loop *Arabidopsis* DE model. It therefore contains the DE model's core LHY–TOC1 negative feedback loop, which was based on an experimental study that demonstrated the repression of TOC1 by LHY while also inferring the activation of LHY by TOC1 [69]. Significantly, the other architecture—which gave the best fit to data—agreed with more recent biochemical work showing that TOC1 is in fact a repressor of LHY [51]. This optimal architecture is also consistent with a complementary computational study showing that an expanded version of the 3-loop DE model incorporating a negative TOC1–LHY interaction yields more accurate simulations of TOC1 knockout and overexpression mutants [70].

We also found that although the cost function used to fit synthetic data only assessed goodness-of-fit in simulated 12:12 LD cycles, the logic models gave very good fits to the DEs in long and short days also, closely reproducing the relationships between photoperiod and expression phase (figure 7). The coordination of biochemical activity with the timing of dawn and dusk is a key system-level property that can be used to assess the relationship between the structure of a circadian network and its evolutionary flexibility [57]. Our photoperiod simulations indicate, somewhat surprisingly, that this property can be accounted for by significantly reduced dynamic models possessing much smaller parameter sets. In particular, the combined dawn- and dusk-tracking observed in the 2-loop *Arabidopsis* DE model (figure 7*c*) is accurately replicated by its logic formulation which has less than one-quarter the number of parameters. A similar reduction was observed for 3-loop *Arabidopsis*, with the 80 parameters describing the DE models decreasing to 20 in its Boolean equivalent (electronic supplementary material, table S1). The ability of simple, discrete models to simulate biologically realistic photoentraiment may also have important implications for the nature of circadian signal processing, in addition to being interesting from a modelling perspective. Specifically, it is consistent with hypotheses based on experimental data suggesting that the mechanism by which daylength and light intensity information is transmitted to output pathways may be partly digital in nature [71].

It should be noted that despite the success of the logic models in reproducing light responses, the coarser representation of network dynamics they provide means that the reproduction of certain circadian properties poses problems for the Boolean approach. A particular restriction of note relates to the modelling of temperature compensation. Temperature can be readily introduced into any DE model by following the methodology originally introduced by Ruoff [72,73]. In this scheme, the temperature dependence of a kinetic parameter is assumed to be governed by the Arrhenius relation. This leads to a simple balance equation for the corresponding activation energies and control coefficients which, when satisfied, guarantees a near-zero period-temperature derivative at the balance point. In a Boolean model, the parameters that determine the free-running period *τ*_FR_ are the discrete delays *τ*_*j*_, and so simple balance equations can be derived from the form of the function determining the dependence of *τ*_FR_ on the *τ*_*j*_s. For example, in the 2-loop *Neurospora* model, it can be shown that *τ*_FR_ = *τ*_1_ + *τ*_2_ + *τ*_3_ + *τ*_4_. Thus, compensation can be achieved simply by choosing the temperature dependence of each delay *τ*_*j*_, so that their sum is constant at each point over the range of interest. However, as the *τ*_*j*_s are generic parameters which in each instance summarize several biochemical processes, any balance equation derived in this manner will not in general be grounded in physical chemistry, reducing its biological relevance.

### 3.2. Computational advantages of the Boolean formulation

In addition to providing a more compact parametrization, a significant strength of the logic modelling approach is that it greatly reduces the complexity of the optimization procedure itself. This enables optimal configurations and delay–threshold combinations to be distinguished with a greater objectivity. The critical simplification afforded by the Boolean formulation is that the cost score is computed from bitstrings, which take the value 0 or 1, rather than data that vary over a much broader range of values. This removes the need for *ad hoc* cost function terms for normalizing the data and robustly computing period and phase. Indeed, the cost function we employed in this work was extremely simple, computing the correlation between the predictions of the model and the corresponding discretized experimental data at each vertex.

A second important simplification relates to the structure of the parameter space. In DE models, each interaction delay is dependent on processes that occur over a range of time scales (e.g. transcription, translation, degradation, etc.). This means that as well as being uncountably infinite, the parameter space can cover several different orders of magnitude, making it difficult to establish *a priori* bounds. In contrast, each signalling delay in a logic model is constrained to be a multiple of the data sampling interval *t*_S_, and is bounded above by the maximum possible time *t*_MAX_ over which the corresponding interaction can occur. *t*_MAX_ is itself bounded by the duration of the experimental measurements and can be further restricted on the basis of biological knowledge (here, the free-running period was used to bound all delays—see §4 for details). The set of possible signalling delays is thus finite. The set of possible discretization thresholds is also finite as varying the threshold for a given species will generate a set number of different binary time series, meaning it is only necessary to consider thresholds for which distinct bitstrings are obtained.

For logic models, the parameter space as a whole is therefore finite, and can be objectively bounded. Furthermore, for a fixed abstract topology, the set of all possible LCs is also finite; it is simply equal to the set of possible Boolean functions. This means that it is, in principle, possible to comprehensively search across *all* possible regulatory structures and parameter combinations to determine the best fit to data. Such a search is impossible with DE systems. Furthermore, searching across different patterns of activation and inhibition for a DE model is often problematic as it requires some *a priori* assumptions to be made regarding the underlying biochemical mechanisms; e.g. specification of which reactions may be cooperative and the ranges of the corresponding Hill coefficients [18,25,34–36]. In practice, however, the number of possible network and parameter combinations in a Boolean formulation can become too large for a complete search with the computational resources available, making it necessary to constrain the optimization.

### 3.3. Refining the optimization protocol

Here, in order to ensure computational tractability, we subsampled the delay–threshold space, while also fixing the parameters controlling the impulse light inputs. In addition, we restricted ourselves to a subset of logic circuits by assuming that (i) multiple light inputs to a gene are combined with an OR gate and (ii) the net light signal directly modulates the state of the gene through either an AND or an OR gate, depending on the free-running light regime (figure 2). Of course not all possible circuits will be biologically reasonable ones (e.g. any circuit for which gates with light inputs uniformly output 0 or 1 would be unviable). Nonetheless, it is reasonable to expect that some interactions may be more accurately modelled by gates that are not of the simple AND/OR type [64], meaning that better performing LCs may have been overlooked.

In view of these restrictions, the fact that our optimal Boolean circuits match the dynamics of their target datasets, both synthetic and experimental, is very encouraging. Indeed, we anticipate that it should be possible to find circuit configurations and parameter sets giving good fits to data over a broader range of genetic and environmental perturbations. For example, our Boolean 3-loop *Arabidopsis* model shows consistency with much of the photoperiodic behaviour of its DE counterpart (figure 7*d*). However, it does not reproduce the phase response observed in the DE model as photoperiod is decreased, for which some components switch between dawn- and dusk-dominance in a complex manner [57].

A probable contributing factor is that our current optimization method involves computing the score at every parameter combination over a fixed lattice. This can be inefficient, particularly where the stoichiometry of an interaction and/or its molecular dynamics require the density of interacting species to accrue beyond a certain value before the interaction is statistically likely to occur. In such cases, the optimal threshold choice for the discretization is likely to be found within a narrow band of possibilities. Furthermore, high threshold resolutions can be required to resolve topological degeneracies in the cost function which make optimal networks difficult to distinguish (see the electronic supplementary material, §S1.2). The accuracy of the optimization could thus be increased by employing logic variants of global search methods capable of providing a more computationally efficient exploration of the parameter space, such as evolutionary algorithms [74]. This would increase the predictive power of the models, better positioning us to address features such as the dawn and dusk switching in shorter photoperiods observed in the 3-loop model.

### 3.4. Future directions

Finally, we note that there are many promising avenues for further developing the approaches introduced in this study. From a theoretical perspective, there is scope for extending established methods for analysing Boolean models. Of particular interest are techniques for determining the simplest inequalities involving linear combinations of the time delays that are required to traverse a given path through the state–space [44,52,53]. In the context of circadian models, this would involve developing general methods for deriving linear inequalities that result in free-running cycles with the target period. As the computational demands of optimization grow exponentially with the number of parameters to be fitted, restricting the search to the corresponding solution set would be expected to dramatically reduce the computational load.

More generally, hybrid logic/DE algorithms would see Boolean methods used firstly to determine the optimal model configuration from data, and then to identify the regions of parameter space within which an equivalent DE model is likely to give a good fit. This would exploit the ability of logic models, demonstrated in this work, to generate a coarse, but quantitative representation of the system dynamics in a systematic and efficient manner. We anticipate that regulatory networks of much greater complexity than those considered here could be quantitively modelled using such an approach.

## 4. Material and methods

### 4.1. Datasets used for model fitting

In order to incorporate some of the variability in expression levels characteristic of real experimental data, synthetic datasets were generated using the variant of Gillespie's stochastic simulation algorithm (SSA) introduced by Gonze *et al.* [39]. Fits to deterministic data obtained from direct integration of the DEs gave very similar results (data not shown). However, the stochastic datasets yielded a clearer separation between LCs, most likely owing to the lifting of topological degeneracies. For all circuits, final analyses were therefore restricted to the results obtained with stochastic time courses.

In generating the synthetic time courses, the scaling (or extensivity) parameter *Ω* was set close to the minimum value yielding self-sustained, unforced oscillations in each case. The final values used for simulations were 1-loop *Neurospora*, *Ω* = 25; 2-loop *Neurospora*, *Ω* = 50; 2- and 3-loop *Arabidopsis*, *Ω* = 1000. Time series were generated for five cycles in both entrained (LD 12:12) and free-running conditions (DD for the *Neurospora* models; LL for *Arabidopsis*). This choice mirrors the combination of experimental datasets typically chosen for parameter optimization in computational circadian studies [18,25,34–36,38]. Time series were then subsampled every 0.5 h to give the data used for model fitting. Plots of the synthetic LD datasets are shown in the electronic supplementary material, figure S5.

Experimental gene expression levels were measured using LUC imaging, carried out as previously described [16]. Images were recorded using Hamamatsu C4742-98 digital cameras operating at −75°C under the control of Wasabi software (Hamamatsu Photonics, Hamamatsu City, Japan) with a sampling interval of 1.5 h. Bioluminescence levels were quantified using Metamorph software (MDS, Toronto, Canada). The resulting expression profiles were detrended for amplitude and baseline damping using the mFourfit function of BRASSv3 [35]. The detrended time series can be seen in figure 9*a*.

### 4.2. Implementing circadian networks in Boolean logic

For *n* biochemical species, let *X*_*i*_(*t*) ∈ {0,1} denote the activity of species *i* at time *t*, where *t* is a multiple, *kt*_S_, of the sampling interval *t*_S_. The update rule is then expressed as4.1
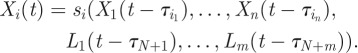
Here, the *L*_*k*_s represent external light inputs to the circuit and *τ*_1_, … , *τ*_*N+m*_ are the signalling delays, with 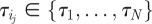 for all *i*,*j*. Assuming a 24 h day and writing *t*_DAWN_ and *t*_DUSK_ for the times of dawn and dusk, respectively, the *L*_*k*_s are described by the following function:4.2
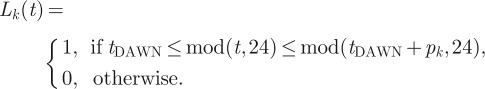
Setting *p*_*k*_ = 0 creates a constant darkness signal (DD); setting *p*_*k*_ = 24 corresponds to constant light (LL); setting *p*_*k*_ = *t*_DUSK_ − *t*_DAWN_ yields a continuous LD cycle. Parameter sets with *p*_*k*_ < *t*_DUSK_ − *t*_DAWN_ yield LD cycles with a pulse of length *p*_*k*_ at dawn.

The functions *s*_*i*_ are Boolean functions *s*_*i*_ : {0,1}^*n+m*^ → {0,1} representing the interactions between genes and light inputs that determine the state of species *X*_*i*_. We introduce a *logical dependency* function (or *logic gate*) *G*(·,*g*) to describe these interactions. This function takes a single parameter *g* ∈ {0,1}, the value of which determines the type of reaction modelled. Formally, we consider two types of operator. The first operator acts on a single Boolean input *Y* ∈ {0,1}, implementing either the identity or NOT gate, modelling activation and repression, respectively:4.3
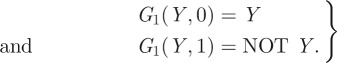
The second type of operator acts on two Boolean inputs *Y*,*Z* ∈ {0,1}, implementing either the AND or the (inclusive) OR dependency. If either species can fulfil the interaction, an OR dependency is used. If both species are required in the interaction, then an AND dependency is used. Thus, for species *Y* ∈ {0,1} and *Z* ∈ {0,1}4.4
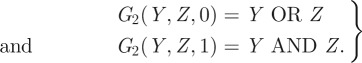


The functions *s*_*i*_ in (4.1) are formed as compositions of these dependencies. For example, the update rule for gene TOC1 in the optimal 2-loop *Arabidopsis* model has the form4.5
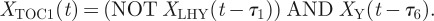
Using (4.3) and (4.4), this can be written as a composition of logic functions4.6
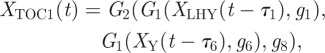
with *g*_6_ = 0 and *g*_1_ = *g*_8_ = 1. The resulting Boolean function models a reaction in which LHY must be downregulated in order for Y to activate TOC1 production.

A given set of interaction functions *s*_*i*_ has an associated adjacency matrix **A** = (*A*_*ij*_) defined by4.7
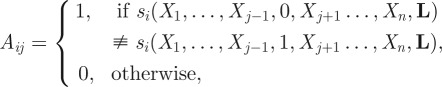
where **L** = (*L*_1_, … , *L*_*m*_). Thus, *A*_*ij*_ = 1 if species *X*_*j*_ can change the state of *X*_*i*_, and so matrix **A** describes the abstract topology of a model (these topologies can be seen in figure 2). It follows that a circadian clock model is parametrized by its adjacency matrix **A**, a set of delays *τ* = (*τ*_1_, … ,*τ*_*N+m*_) and a set of gates **G** = (*g*_1_, … , *g*_*d*_). Writing **X** = (*X*_1_, … , *X*_*n*_), this yields the following compact, vectorized form of the update rule:4.8
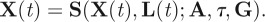
The set of gates **G** is the model's logic configuration (LC). As each configuration **G** = (*g*_1_, … , *g*_*d*_) is a bitstring, it can be represented uniquely by the corresponding decimal expansion *D*(**G**) defined as4.9
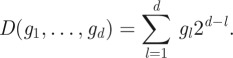
For the models considered, the LCs are enumerated in terms of their decimal expansions for simplicity in figures 4, 5 and 8.

Of the LCs consistent with **A**, a subset matches the pattern of activation and inhibition in the corresponding DE model. These are referred to as the DE LCs. For example, for the 2-loop *Neurospora* model, *frq* activates both isoforms of FRQ, giving *g*_1_ = *g*_2_ = 0, and both isoforms repress transcription, giving *g*_3_ = *g*_4_ = 1 (figures 1*b* and 2*b*). There are thus a pair of DE LCs in this case, 00110 and 00111, depending on whether both isoforms are required for repression (*g*_5_ = 0, corresponding to OR) or a single isoform is sufficient (*g*_5_ = 1, corresponding to AND). These DE LCs are encoded by the integers 6 and 7, respectively.

Finally, in order to construct the simplest logic models consistent with the general form of circadian DE systems, each Boolean function *s*_*i*_ is assumed to have the form4.10
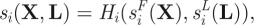
such that (i) *H*_*i*_ : {0,1}^2^ → {0,1} implements either the AND or OR gate; (ii) *s*_*i*_^*F*^ : {0,1}^*n*^ → {0,1} encodes the structure of the free-running system; and (iii) *s*_*i*_^*L*^ : {0,1}^*m*^ → {0,1} determines how multiple light inputs are integrated with *s*_*i*_^*L*^(0, … , 0) = 0 and *s*_*i*_^*L*^(1, … , 1) = 1. For consistency, it is therefore necessary that setting each light input *L*_*k*_ to the relevant constant value *L* recovers the free-running system (*L* = 0 for DD; *L* = 1 for LL). This condition is equivalent to *H*_*i*_(*s*_*i*_^*F*^(**X**),*L*) = *s*_*i*_^*F*^(**X**). For the *Neurospora* models, where the free-running condition is DD, the consistency condition is achieved using an OR gate because *x* OR 0 = *x*. In the case of the *Arabidopsis* circuits, where the free-running condition is LL, the AND gate is appropriate as *x* AND 1 = *x*.

For both *Arabidopsis* models, the multiple light inputs to Y are combined using an OR gate: this is because in the corresponding DE models, both inputs can independently upregulate transcription [35,36]. In addition, the 3-loop model incorporates only the pulsed light input to the PRR gene because removing the continuous light input from the DE system had a negligible effect on its photoperiodic behaviour.

Full details of the logic formulation of each clock network are given in the electronic supplementary material, §S2.

### 4.3. Optimization and constraints

In order to identify the optimal combination of signalling delays *τ* = (*τ*_*j*_), thresholds **T** = (*T*_*i*_) and logic gates **G** = (*g*_*l*_) for a given model and dataset, we must introduce a cost function to be minimized. We used a simple function based on a correlation between the predicted time courses generated by the model and the corresponding data. Further details can be found in the electronic supplementary material, §S1.1. Optimal LC–parameter combinations are given in tables 1 and 2.

**Table 1.** Optimal parameter sets: synthetic data. The logic configurations, **G**, delays, *τ*_*j*_, and discretization thresholds, *T*_*i*_ (0 < *T*_*i*_ < 1), yielding the best fit of each logic model to synthetic time series. The values used for photoperiod simulations—obtained by fitting directly to DE time series—are shown in brackets. For each model, *p*_*k*_ indicates the parameter used to simulate light input *L*_*k*_ through equation (4.2). These were fixed at the values shown, with *P* denoting the photoperiod *t*_DUSK_ − *t*_DAWN_.

	1-loop *Neurospora*	2-loop *Neurospora*	2-loop *Arabidopsis*	3-loop *Arabidopsis*
**G**	01	00111	10011011	10011011011
*τ*_1_ (h)	5 (5)	5 (5.5)	1.5 (3)	0 (3)
*τ*_2_ (h)	6.5 (6.5)	1.5 (2)	5.5 (6)	5.5 (6.5)
*τ*_3_ (h)	7.5 (9)	6 (6.5)	6.5 (7.5)	7 (8)
*τ*_4_ (h)	—	10 (9)	0 (0.5)	0 (1)
*τ*_5_ (h)	—	9 (9)	7.5 (6)	8 (5)
*τ*_6_ (h)	—	—	4 (4)	5 (3.5)
*τ*_7_ (h)	—	—	0 (1)	4.5 (6)
*τ*_8_ (h)	—	—	2.5 (0.5)	6 (5)
*τ*_9_ (h)	—	—	1 (0)	1 (0)
*τ*_10_ (h)	—	—	—	1 (3)
*τ*_11_ (h)	—	—	—	0.5 (0)
*τ*_12_ (h)	—	—	—	3 (4)
*T*_1_	0.35 (0.40)	0.425 (0.425)	0.250 (0.450)	0.1250 (0.4350)
*T*_2_	0.40 (0.70)	0.525 (0.650)	0.375 (0.775)	0.3000 (0.6850)
*T*_3_	—	0.250 (0.350)	0.575 (0.750)	0.6250 (0.7325)
*T*_4_	—	—	0.150 (0.325)	0.2250 (0.1295)
*T*_5_	—	—	—	0.8250 (0.9500)
*p*_1_ (h)	*P*	*P*	2	2
*p*_2_ (h)	—	*P*	*P*	*P*
*p*_3_ (h)	—	—	0.5	0.5
*p*_4_ (h)	—	—	—	3

**Table 2.** Optimal parameter sets: experimental data. The logic configurations (LCs), **G**, delays, *τ*_*j*_, and discretization thresholds, *T*_*i*_ (0 < *T*_*i*_ < 1), yielding the top two fits of the 3-loop *Arabidopsis* logic model to experimental LUC time series recorded in LL. *G*_OPT_ is the highest-scoring LC. *G*_DE_ is the second highest-scoring LC, and is also the top-ranked configuration under the constraint that LHY represses TOC1 and TOC1 promotes LHY production. Note that *G*_DE_ was previously identified as the LC giving the optimal fit to synthetic data generated by the equivalent DE model (cf. table 1).

**G**	10101011011 (*G*_OPT_)	10011011011 (*G*_DE_)
*τ*_1_ (h)	1.5	1.5
*τ*_2_ (h)	0	0
*τ*_3_ (h)	1.5	10.5
*τ*_4_ (h)	9	0
*τ*_5_ (h)	6	7.5
*τ*_6_ (h)	4.5	4.5
*τ*_7_ (h)	1.5	3
*τ*_8_ (h)	10.5	6
*τ*_9_ (h)	0	0
*τ*_10_ (h)	0	0
*τ*_11_ (h)	0	0
*τ*_12_ (h)	0	0
*T*_1_	0.30	0.35
*T*_2_	0.40	0.35
*T*_3_	—	—
*T*_4_	0.15	0.10
*T*_5_	0.50	0.45
*p*_1_ (h)	24	24
*p*_2_ (h)	24	24
*p*_3_ (h)	24	24
*p*_4_ (h)	24	24

The most comprehensive strategy in seeking to minimize the cost function is to systematically calculate the cost for all possible configurations and parametrizations of the model; that is all delays 0 ≤ *τ*_*j*_ ≤ *t*_MAX_, where *t*_MAX_ is the longest time scale considered in the system, and all thresholds 0 < *T*_*i*_ < 1. However, when the model becomes too complex to permit a global analysis, it becomes necessary to introduce further constraints to restrict the parametrization to be considered. Such constraints should be as objective as possible.

One viable, objective constraint is to limit all the signalling delays around a closed loop so that they sum to no more than the period *τ*_FR_ of free-running oscillations in the target dataset.

For 1-loop *Neurospora*, this gives the single delay bound4.11
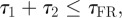
where *τ*_FR_ = 22 h.

For 2-loop *Neurospora*, the constraint results in two delay bounds4.12
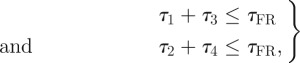
where again *τ*_FR_ = 22 h.

For 2-loop *Arabidopsis*, there are three delay bounds4.13
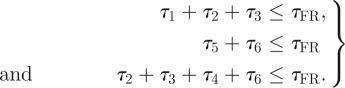
In this case, *τ*_FR_ = 25 h.

For 3-loop *Arabidopsis*, there are four delay bounds: those for the 2-loop model above together with an extra bound introduced by the addition of the LHY–PRR loop:4.14
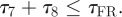
For this circuit, *τ*_FR_ = 24 h.

Two further constraints were introduced for reasons of computational tractability. Firstly, each delay *τ*_*j*_ was restricted to integer multiples of a minimum delay resolution *τ*_*R*_, itself a multiple *k*_*τ*_*t*_S_ of the data sampling interval *t*_S_. Secondly, each threshold *T*_*i*_ was bounded within a subinterval [*T*_MIN_,*T*_MAX_] of [0,1], and also restricted to integer multiples of a minimum threshold resolution *T*_*R*_.

#### 4.3.1. Optimization to synthetic data

For all models, *T*_MIN_ and *T*_MAX_ were set to the values 0.2 and 0.8, respectively. For the *Neurospora* models, *k*_*τ*_ was set to 1 and *T*_*R*_ to 0.025; for the *Arabidopsis* models, *k*_*τ*_ was initially set to 2 and *T*_*R*_ to 0.05. Scores were then recalculated with *k*_*τ*_ = 1 and *T*_*R*_ = 0.025, within intervals [*τ*_*i*_ − 3*τ*_*R*_,*τ*_*i*_ + 3*τ*_*R*_] and [*T*_*j*_ − 5*T*_*R*_, *T*_*j*_ + 5*T*_*R*_] centred around the parameter combinations giving the best scores. For *Arabidopsis* optimizations, light parameters controlling the impulse inputs (*L*_1_ and *L*_3_ in the 2-loop circuit; *L*_1_, *L*_3_ and *L*_4_ in the 3-loop circuit) were fixed at the values shown in table 1. These were determined from discrete approximations to the corresponding continuous curves in the DE models.

Each parameter set was then checked for a functioning circadian clock. Firstly, the solution to the free-running model was generated under the appropriate continuous light conditions, using the discretized data as initial conditions. If a limit cycle was obtained with period within 20% of the DE model, then this was fed into the model under simulated 12:12 LD cycles. Periodic, entrained oscillations were taken to indicate a viable clock circuit.

For the *Neurospora* and 2-loop *Arabidopsis* models, optimizations included all possible LCs. The 3-loop *Arabidopsis* optimizations were restricted to the subset of LCs defined by **G** = (10011011*xyz*), *x*,*y*,*z* ∈{0,1}; these are the configurations that result from fixing gates *g*_1_, … , *g*_7_ to their optimized values in the 2-loop circuit.

The photoperiod simulations shown in figure 7 were obtained by locally re-optimizing the logic circuits yielding viable clocks to simulations of the DE models under 12:12 LD cycles, and then calculating the maximum symmetric photoperiod interval (12 − *P*_MAX_, 12 + *P*_MAX_) over which both model formulations generated stable, entrained solutions. The cost was minimized with a simulated annealing algorithm [18,34,75], using a cost function for which the data and predicted time courses were taken to be single cycles of the entrained solution in the DE and logic models, respectively.

#### 4.3.2. Optimization to experimental luciferase data

In fitting the 3-loop *Arabidopsis* model to the LUC data shown in figure 9*a*, genes were matched to model variables in the following manner: (i) CCA1 was equated to LHY on the basis that the LHY variable in the equivalent DE model groups together the effect of the two genes [36]; (ii) TOC1 was matched to TOC1; (iii) GI was matched to Y owing to experimental results showing that this gene can account for some of the action of Y in the DE model [36]; and (iv) PRR9 was matched to PRR, as this variable combines the PRR7 and 9 genes in the DE formulation [36].

Because the exact biological correlate of variable X is currently unknown, it was not used for costing the fit. Consequently, *g*_2_ and *τ*_2_ were both fixed at 0 (note that fixing *g*_2_ reduces the number of possible LCs from 2048 to 1024). This preserves the dynamics of all components in the model excluding X, together with the delay bounds used for constraining the parameter space. It also means that TOC1 and the dummy variable X have identical time series. Thus, in practice, the discretized TOC1 expression time series were used as a proxy for X data when calculating the cost at the LHY vertex. In addition, as the LUC data were measured in LL, the parameters *p*_1_ → *p*_4_ controlling the light inputs were all set to 24 (cf. equation (4.2)). In LL conditions, the absence of dawn and dusk means that the choice of the delay parameters *τ*_9_ → *τ*_12_ is arbitrary. We therefore set the values of these delays to 0. *T*_MIN_ and *T*_MAX_ were fixed at 0.2 and 0.8, respectively. *k*_*τ*_ was initially set to 2 and *T*_*R*_ to 0.2. Scores were then recalculated with *k*_*τ*_ = 1 and *T*_*R*_ = 0.05, within intervals [*τ*_*i*_ − 2*τ*_*R*_,*τ*_*i*_ + 2*τ*_*R*_] and [*T*_*j*_ − 2*T*_*R*_,*T*_*j*_ + 2*T*_*R*_] centred around the best-scoring parameter sets. The optimal parameter set for each LC was assessed to determine whether it yielded a viable clock by first generating the solution to the model using the discretized LUC time series as initial conditions, and then checking that this gave a limit cycle with free-running period within 20% of 24 h.

### 4.4. Software

The numerical routines for parameter optimization and model simulation were initially developed in Matlab (Mathworks, Cambridge, UK) and C. The scoring algorithms used for global parameter sweeps were subsequently converted into Java and run on a task farm computer consisting of 118 Intel Harpertown quad-core processors. All software used is available on request from the corresponding author.
